# Multicenter study of colon capsule endoscopy in post-polypectomy surveillance

**DOI:** 10.1055/a-2779-1774

**Published:** 2026-01-13

**Authors:** James Turvill, Monica Haritakis, Scott Pygall, Emily Bryant, Harriet Cox, Greg Forshaw, Crispin Musicha, Victoria Allgar, Robert Logan, Mark McAlindon

**Affiliations:** 1 8749 Gastroenterology, York and Scarborough Teaching Hospitals NHS Foundation Trust, York, United Kingdom of Great Britain and Northern Ireland; 2 8749 Research and Innovation, York and Scarborough Teaching Hospitals NHS Foundation Trust, York, United Kingdom of Great Britain and Northern Ireland; 3 556540 Improvement Delivery, NHS England and NHS Improvement East of England, Welwyn Garden City, United Kingdom of Great Britain and Northern Ireland; 4 2394 NHS Cancer Programme, NHS England, Redditch, United Kingdom of Great Britain and Northern Ireland; 5 6633 Medical Statistics Group, Plymouth University, Plymouth, United Kingdom of Great Britain and Northern Ireland; 6 2394 Department of Gastroenterology, King's College London, London, United Kingdom of Great Britain and Northern Ireland; 7 7318 Academic Unit of Gastroenterology and Hepatology, Sheffield Teaching Hospitals NHS Foundation Trust, Sheffield, United Kingdom of Great Britain and Northern Ireland

**Keywords:** Endoscopy Lower GI Tract, Colorectal cancer, Polyps / adenomas / ..., Quality and logistical aspects, Performance and complications

## Abstract

**Background and study aims:**

During the COVID-19 pandemic the National Health Service introduced colon capsule endoscopy (CCE) as an alternative to colonoscopy in patients awaiting 3-year post-polypectomy surveillance. We determined the safety, diagnostic accuracy, and utility of CCE in this clinical setting.

**Patients and methods:**

Consenting patients awaiting 3-year post-polypectomy surveillance underwent CCE or colonoscopy. For those having CCE, risk-based guidance was developed directing to: 1) immediate colorectal endoscopic intervention; 2) deferred intervention; or 3) discharge. The safety, comparative and paired diagnostic accuracy, and colonoscopy capacity spared by CCE were determined.

**Results:**

There were 464 CCE and 78 colonoscopy patients recruited. CCE patients were younger (mean 62 years versus 68 years). CCE was safely tolerated in 99% of patients. More ≥ 10 mm and 6- to 9-mm polyps were detected in the CCE cohort than the colonoscopy comparator cohort. This was on an intention to investigate basis and in those who had complete and adequately prepared examinations. Two hundred and five CCE patients had an urgent colonoscopy or flexible sigmoidoscopy and their paired findings were matched. Per patient sensitivities for ≥ 10 mm and 6- to 9-mm polyps were 92% and 90%, respectively. Two-thirds of patients entered a modified management pathway after CCE with 25% being discharged and 27% having a procedure deferral for up to 3 years. CCE completion and bowel preparation adequacy rates were 78% and 73% respectively. No colorectal cancer was detected.

**Conclusions:**

CCE is a safe diagnostic of colorectal polyps. In surveillance, its "filter function" complements existing colorectal diagnostic services by providing capacity and choice.

## Introduction

In 2022, during the recovery phase of the COVID-19 pandemic, the National Health Service (NHS) England National Cancer Team recognized that the continued pressure on urgent care in endoscopy services was placing those awaiting surveillance colonoscopy at risk 1 2 . In response to this, colon capsule endoscopy (CCE) was introduced to provide additional capacity for patients due or overdue for a post-polypectomy surveillance colonoscopy. There was an existing evidence base for the safety and diagnostic accuracy of CCE in a surveillance population 3 4 . The case was made that introducing CCE as a diagnostic filter test would permit personalized risk assessment for patients. Accepting that the CCE may not be definitive, it might identify: 1) some who could be discharged; and 2) others who required a therapeutic intervention, the timing and nature of which could be tailored to the patient and pathology. Colonoscopy capacity could then either be spared or better managed and, more importantly, patients could be safely advised 5 6 .

**Fig. 1:**
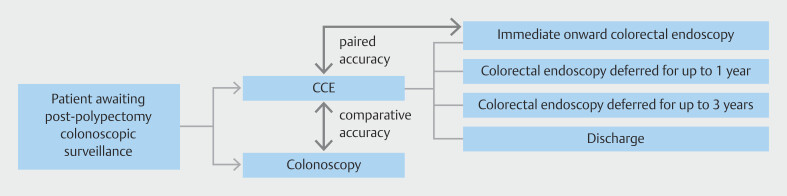
Schematic of the study design.

The aim of this study was to determine, prospectively, the safety and diagnostic accuracy of CCE in this clinical setting and to assess the impact of CCE on colorectal endoscopic capacity in the surveillance population. Both paired and comparative assessments of diagnostic accuracy were to be made ( Fig. 1 ).

## Patients and methods

### Patients and study design

Inclusion criteria were consenting patients aged 18 years or older, due or overdue 3 yearly post-polypectomy surveillance colonoscopy and who underwent either CCE or colonoscopy 7 . Compliance with the 2020 guidelines for post-polypectomy surveillance was recommended but not mandated, noting that each site in the study was responsible for managing its own post-polypectomy surveillance population. Exclusion criteria were patients in other surveillance groups, notably patients awaiting inflammatory bowel disease surveillance, Lynch syndrome patients, 1- or 3-year post-colorectal cancer surgery surveillance patients or patients who were awaiting a site check post large, and non-pedunculated colorectal polyp removal because there was an inadequate evidence base to support this.

Noting the clinical context in which CCE was being introduced and the need to create additional colorectal diagnostic capacity, a pragmatic study design was developed, permitting the diagnostic accuracy of CCE to be assessed in two ways ( Fig. 1 ). Initially, the diagnoses of two separate cohorts of patients, one undergoing CCE and the other colonoscopy, were to be compared. Because the cohorts were likely to be similar, it was argued that prevalence of colorectal disease would similarly be matched. This is referred to as the comparative accuracy arm of the study. Thereafter, a paired comparison of findings was undertaken in those who went on to colonoscopy (or flexible sigmoidoscopy) after CCE for polypectomy or because the CCE examination was incomplete or the bowel preparation inadequate. This is referred to as the paired accuracy arm.

There were no formal CCE exclusion criteria. Instead, guidance to consider excluding patients at risk of being unable to swallow the capsule, tolerate, or comply with the CCE procedure or who were at risk of small or large bowel stricturing was provided to clinicians by an Expert Advisory Group (EAG) that had been convened by the National Cancer Team. The EAG specifically advised that patients with significant comorbidity, use of opioid or tricyclic antidepressant medication, and impaired mobility may predict for poor bowel preparation. However, choice of test was at the discretion of the responsible clinician at the participating site and the patient.

The EAG developed tiered guidance to support clinicians in the onward management of patients after they had completed their CCE ( Fig. 1 ). It was based on existing British and European polyp surveillance guidance and clinical expert opinion and is summarized below:

Tier 1: High-risk polyps identified at CCE (1 polyp ≥ 10 mm or ≥ 5 polyps): proceed directly to therapeutic colonoscopy or flexible sigmoidoscopy.

Tier 2: Intermediate-risk polyps identified at CCE (1 polyp 6–9 mm or 3–4 polyps): offer therapeutic colonoscopy or flexible sigmoidoscopy deferred for up to 1 year.

Tier 3: Low-risk polyps identified at CCE (< 3 polyps all < 6 mm): surveillance colonoscopy at 3 years.

Tier 4: No polyps identified: discharge 8 9 .

When the CCE was either incomplete or inadequately prepared, the clinician was expected to proceed to colonoscopy or flexible sigmoidoscopy as clinically indicated. Colonoscopy was performed and reported according to the practice of each individual center with the Boston Bowel Preparation Scale being applied 10 . CCE was performed using the PillCam COLON 2 system (Medtronic.com) 11 . All centers followed the guidelines of the European Society of Gastrointestinal Endoscopy (ESGE) for CCE bowel preparation 8 . A split-dose regimen of polyethylene glycol-electrolyte solution was used (Moviprep (Norgine Ltd) or Plenvu (Norgine Ltd) (when stocks were depleted). The two directed “boosters” were gastrografin and phosphosoda (or phosphosoda alone when gastrografin could not be accessed) and if needed, a bisacodyl suppository was used. A complete CCE study was defined as one where the CCE was seen to be expelled or where the anal cushions were identified and an adequately prepared CCE achieved a score ≥ 6 on the Colon Capsule Clear Score 12 . All CCE video reporters had completed an approved online CCE training course (Imige Ltd) 13 14 .

### Data collection

Patient details were anonymized using a personal identification number assigned by the local centers. All data were entered by the local research team in each participating center into an electronic case report form. For all procedures this included demographic data, details of the index investigation, and the indication for the surveillance procedure. Also required was a description of all pathology identified and an estimation of size and site (right, transverse or left colon or rectum) of any mass lesions detected, a description of the completeness and adequacy of examination, and any complications arising. Complications and existing quality and safety standards for colonoscopy were reported 15 .

Details about patients who went on to immediate colorectal endoscopy were recorded to permit determination of paired accuracy. This largely includes Tier 1 patients and those with an incomplete CCE or where the bowel preparation was inadequate. Outcomes of other tier patients awaiting follow up are not reported here. Recognizing that “false-negative” patients could be missed in this paired accuracy cohort, the parallel comparative study arm permitted a comparative assessment of clinical findings from the separate “matched” colonoscopy cohort.

### Powering

A powering calculation was developed based on the comparative nature of the initial study design. Noting that this was a 1:1 study comparing CCE with colonoscopy with the primary variable being detection of polyps ≥ 6 mm in size, it was estimated that to detect a 10% relative reduction in significant polyp detection would require 8084 patients in each of the two cohorts. Unfortunately, due to the termination of the NHS study, recruitment to this target could not be achieved and a statistical analysis based on this design has not been performed. Instead, the data are presented descriptively.

### Statistical analysis

Data are presented descriptively as mean (with standard deviation) or median (with interquartile ranges). A *t* -test was used to compare the comparator populations as appropriate. Polyp matching was undertaken in line with that described by Rex et al 16 . In brief, for a polyp detected at CCE to be considered a true positive, it had to match a polyp found at endoscopy being: 1) located within the same or adjacent colonic segment (right, transverse or left colon and rectum); and 2) in the same or adjoining size range as found at endoscopy. Polyps reported by CCE but not matched at endoscopy were regarded as false positives. Polyps detected by endoscopy but not reported by CCE were regarded as false negatives. Matching was also reported on a per-patient basis. Here the polyps were matched by the same size methods but without reference to the colonic segment and, when multiple polyps, patients were regarded as true positives only if they all were true-positive polyps. If there were any false-negative polyps, the patient was regarded as a false negative.

### Trial monitoring group

Funding by NHS England National Cancer Programme to support its introduction was a pragmatic response aimed at ameliorating pressures on endoscopy services during and following the COVID-19 pandemic. Therefore, data submitted were regularly reviewed by the EAG to evaluate and compare progress in each participating center and monthly meetings were held with frontline staff to share best practice.

Ethical approval was obtained to conduct this study: IRAS ID: 156515 and all patients provided written, informed consent to participate.

### Outcomes

The primary outcomes of the study were to determine the safety and diagnostic accuracy of CCE for colorectal cancer and high-risk polyps (those ≥ 6 mm) in 3-year post-polypectomy surveillance. Secondary outcomes were to determine the colonoscopy capacity spared by CCE when findings were stratified by risk. A health economic assessment was not undertaken.

## Results

### Recruitment, CCE performance and safety

From March 2022 to March 2024, a total of 464 patients having CCE were recruited from 31 NHS Trusts in England. Polyps had been reported in 96% of patients at their index procedure, but compliance with the updated British Society of Gastroenterology/Association of Colo-Proctology of Great Britain and Ireland (BSG/ACPGBI) 3 yearly post-polypectomy surveillance criteria was only 45% (10% ≥ 5 polyps and 39% ≥ 2 premalignant polyps with ≥1 advanced polyp) 7 . The mean age was 62 (± 10) years and 41% were female. A patency capsule was used in only eight patients and the complication rate was 1%: one patient vomited the capsule and four had suspected retention, requiring abdominal x-ray. One capsule was a confirmed retention in a sigmoid diverticular segment at the time of reporting. There were also three technical failures and two patients had ultra-rapid transit. CCE was complete in 79% of men and 78% in women, 77% for patients ≥ 60 years and 81% in those < 60 years. A CC-Clear score was ≥ 6 in 73% of patients giving a complete and adequately prepared bowel in 62% of patients. Of the 100 incomplete procedures, 36 obtained satisfactory views of the right and transverse colon, making access to the unexamined colon via flexible sigmoidoscopy an option. Positive pathology was identified in 398 patients (86%) at CCE ( Table 1 ). No patients had colorectal cancer but 987 polyps (median 3) were found among 306 CCE patients (66%). A similar proportion of all polyps, 6- to 9-mm, and ≥ 10-mm polyps, and the polyp-to-patient ratio was seen in the 289 CCE patients with complete and adequately prepared procedures ( Table 2 ).

**Table:** **Table 1** Pathology found at CCE and colonoscopy, each as the index surveillance investigation and on an intention-to-investigate basis.

Characteristic	CCE	Colonoscopy
	n = 464 (%)	n = 78 (%)
CRC	0	0
Patients with polyps		
all polyps	306 (66)	46 (58)
≥ 10 mm	95 (20)	9 (11)
6–9 mm	185 (40)	19 (25)
≥ 6 mm	218 (47)	19 (24)
≥ 3 polyps	155 (33)	23 (29)
Polyp numbers and site		
all polyps	987	159
≥ 10 mm	127 (13)	9 (6)
Right colon	42 (33)	5 (56)
Transverse colon	29 (23)	1 (11)
Left colon	45 (35)	3 (33)
Rectum	11 (9)	0
6–9 mm	346 (35)	19 (24)
Right colon	93 (27)	12 (63)
Transverse colon	72 (21)	1 (5)
Left colon	152 (44)	5 (26)
Rectum	29 (8)	1 (5)
≥ 6 mm	473 (48)	28 (18)
Polyp to patient ratio		
≥ 10 mm	1.3	1.0
6–9 mm	1.9	1.0
Colitis	2 (0.4)	0
Diverticulosis	255 (55)	24 (31)
Diverticulitis	3 (0.8)	0
Other gastrointestinal diseases	99 (21)	6 (8)

**Table:** **Table 2** Pathology found at CCE and colonoscopy, each as the index surveillance investigation in those with complete and adequate studies.

Characteristic	CCE	Colonoscopy
	n = 289 (%)	n = 49 (%)
Normal	40 (14)	7 (14)
CRC	0	0
Patients with polyps		
all polyps	194 (67)	32 (65)
≥ 10 mm	54 (19)	5 (10)
6–9 mm	109 (38)	12 (24)
≥ 6 mm	130 (45)	11 (22)
≥ 3 polyps	95 (33)	16 (33)
Polyp numbers and site		
all polyps	624	108
≥ 10 mm	72 (12)	5 (5)
6–9 mm	212 (34)	12 (12)
≥ 6 mm	284 (46)	17 (17)
Polyp to patient ratio		
≥ 10 mm	1.3	1.0
6–9 mm	1.9	1.0

### Paired CCE diagnostic accuracy

Matching of outcomes was performed on 205 paired patients (44%) who went on to immediate colonoscopy (159; 34%) or flexible sigmoidoscopy (46; 10%) after CCE. Men constituted 58% of this group and 65% were ≥ 60 years. Data are also available on a further four patients that had been coded for a deferred investigation or discharge but who had a colonoscopy during the study ( Table 3 ). Polyps were identified in 79% of CCE patients but 67 patients (32%) required onward colorectal endoscopy because an incomplete or inadequately prepared CCE. Recorded bowel preparation at colonoscopy was good or excellent in 90% of patients, the cecal intubation was 96% and mean extubation time was 12 minutes. Endoscopy was normal in 18% of patients but more polyps overall and polyps 6 to 9 mm and ≥ 10 mm, both on a per-polyp and per-patient basis, were identified at CCE compared with the subsequent gold standard. Double the number of patients with a polyp ≥ 10 mm were reported at CCE.

**Table:** **Table 3** Matched pathology findings in patients who had follow-up colonoscopy/flexible sigmoidoscopy after a surveillance CCE.

Characteristic	CCE	Colonoscopy	Flexible sigmoidoscopy	Total
	(n = 209*)	(n = 163)	(n = 46)	(n = 209)
Normal		23 (14)	14 (30)	37 (18)
Patient with polyps				
all polyps	167 (79)	127 (77)	23 (50)	150 (71)
≥ 10 mm	78 (37)	30 (18)	4 (9)	34 (16)
6–9 mm	114 (54)	48 (29)	8 (17)	56 (27)
≥ 6 mm	141 (67)	65 (39)	12 (26)	77 (36)
≥ 3 polyps	97 (46)	74 (45)	6 (13)	80 (38)
≥ 5 polyps	68 (25)	36 (22)	2 (4)	38 (18)
Polyp numbers and site				
all polyps	622	436	47	483
≥ 10 mm	104 (17)	41 (9)	4 (9)	45 (9)
Right colon	34 (33)	16 (39)	0	16 (36)
Transverse colon	23 (22)	11 (27)	0	11 (24)
Left colon	37 (36)	11 (27)	2 (50)	13 (29)
Rectum	10 (10)	3 (7)	2 (50)	5 (11)
6–9 mm	234 (38)	70 (16)	9 (19)	79 (16)
Right colon	63 (27)	29 (41)	0	29 (37)
Transverse colon	45 (19)	17 (24)	0	17 (22)
Left colon	107 (46)	20 (29)	6 (67)	26 (33)
Rectum	19 (8)	4 (6)	3 (33)	7 (9)
≥ 6 mm	338 (54)	111 (25)	13 (28)	124 (26)
Polyp per patient ratio				
≥ 10 mm	1.3	1.4	1.0	1.3
6–9 mm	2.1	1.5	1.1	1.4
Colitis	1 (0.5)	0	1 (2.1)	1 (0.5)
*205 patients who went on to immediate colonoscopy in line with guidance but data are also available on a further four patients that had been coded for a deferred investigation or discharge but who had a colonoscopy during the study period.				

Formal polyp matching was conducted on those who had complete and adequately prepared procedures for polyps ≥ 10 mm and 6 to 9 mm ( Table 4 ). The per-polyp matching sensitivity was 80% to 82% whereas it was 90% to 92% on a per-patient basis.

**Table:** **Table 4** Per polyp and per patient polyp matching.

	Polyp ≥ 10 mm		Polyp 6–9 mm	
	Per patient	Per polyp	Per patient	Per polyp
CCE true positives	34	40	64	108
CCE false positives	55	56	47	94
CCE false negatives	3	10	7	23
Sensitivity % (confidence intervals)	92% (77–98)	80% (66–90)	90% (80–96)	82% (75–89)

#### Onward investigation post CCE and capacity spared

**Fig. 2:**
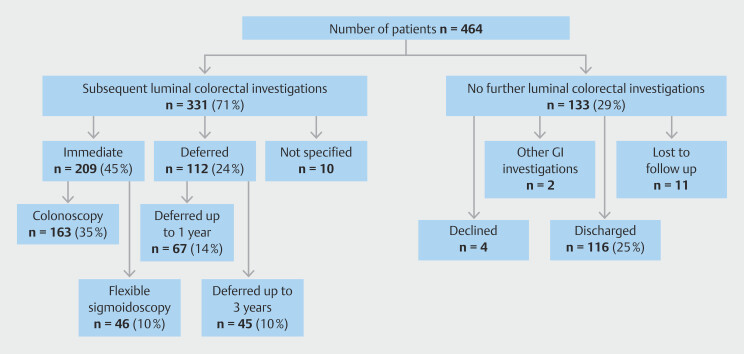
Onward management of surveillance patients post-CCE.

**Fig. 3:**
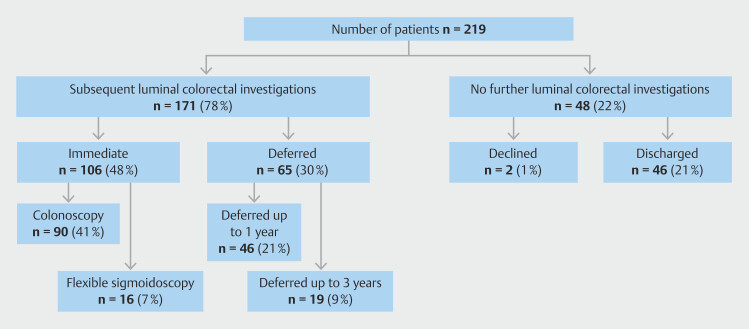
Onward management of compliant 3-year post-polypectomy surveillance patients post-CCE.

As outlined above, 205 (44%) patients went onto to immediate colorectal endoscopy (159; 34%) or flexible sigmoidoscopy (46; 10%) after CCE ( Fig. 2 and Fig. 3 ). Men constituted 58% of this group and 65% were ≥ 60 years. Of the patients, 259 (56%) had a modified outcome in line with the clinical guidance provided:

Tier 2: 78 patients (17%) had an investigation deferred for up to 1 year;

Tier 3: 47 patients (10%) were placed on a 3 yearly surveillance waiting list and

Tier 4: 132 patients (29%) were discharged or no defined outcome decision.

Two patients were described as other.

Of the patients, 353 (76%) were referred for colorectal endoscopy in line with the surveillance CCE polyp management guidelines. There were a further 67 patients in whom the guidelines could not be applied because of an incomplete or inadequately prepared CCE and who went on to endoscopy directly. Of the remaining 44 (9%), the onward management reason was described as clinician choice. Sometimes this resulted in an escalation of colorectal endoscopy for low-risk polyps but also sometimes reflected patient frailty and prompted a lesser investigation or was unexplained. In this way, 19 patients had an immediate intervention and seven and six patients, respectively, did not follow the Tier 2 and 3 guidelines as otherwise directed. A further 12 patients were discharged, six patients had a polyp ≥ 6 mm or ≥ 3 diminutive polyps, but their cases were reviewed and a clinical decision was made to discharge. Six patients declined follow up. No patients have been reported with colorectal cancer during the study

A similar pattern of outcomes was seen in a subgroup analysis of the 219 patients who strictly fulfilled the BSG/ACPGBI 3 yearly post-polypectomy surveillance criteria. However, 48% rather than 44% were referred for immediate colorectal endoscopy and 22% rather than 29% had no further luminal interventions ( Fig. 3 ).

### Comparative accuracy assessment of separate CCE and colonoscopy cohorts

Only 78 patients were recruited into the comparative colonoscopy cohort. It had been argued that, should the CCE patient cohort match the colonoscopy cohort in terms of surveillance indications, age, and sex, then the comparative prevalence of polyps in each cohort should be matched ( Table 1 and Table 2 ). Sex distribution was similar, but the mean age of 68 years (± 10) in those having colonoscopy was significantly higher ( *P* < 0.01). Compliance with BSG/ACPGBI guidelines was 68% (35% ≥ 5 polyps and 46% ≥ 2 premalignant polyps with ≥ 1 advanced polyp) in the comparator colonoscopy cohort. Nonetheless, positive pathology was found in only 57 of those having colonoscopy (73%), with 159 polyps (median 3) in 46 patients (58%). This amounts to more polyps overall and polyps 6 to 9 mm and ≥ 10 mm, both on a per-polyp and per-patient basis, and on an intention-to-investigate basis, and in complete and adequate studies being detected in the CCE cohort compared with the colonoscopy cohort.

## Discussion

This is the largest CCE study to have taken place in a surveillance population. That it has been a multicenter study, recruiting patients within standard clinical care, reinforces its immediate applicability. CCE was well tolerated and had a per-patient diagnostic sensitivity for polyps of 90% to 92% 17 18 19 . Specificity could not be calculated because patients with a normal CCE were discharged 20 . The pragmatic onward triaging of patients into the four risk tiers that had been developed by the EAG worked well, with a clinician compliance > 90% demonstrated. No colorectal cancers were detected or missed in patients awaiting surveillance and who had CCE. Twenty-nine percent of patients had no further colorectal investigation, 27% had a procedure deferred for up to 3 years and only one-third of patients required immediate onward colonoscopy. Of those patients who went on to immediate onward colonoscopy or flexible sigmoidoscopy 71% had either a polyp ≥ 10 mm or ≥ 5 polyps. Accepting that introduction of CCE as a filter test imposed an additional round of bowel preparation and potential inconvenience for those in whom the gold standard and therapeutic colonoscopy was required, CCE modified the onward management of two-thirds of patients. Weighing the needs of surveillance patients known to be at higher risk of colorectal cancer against symptomatic patients within a suspected colorectal cancer pathway can be a challenge for units that have constrained colorectal diagnostic capacity. Here CCE, which can be upscaled without the infrastructure and training demands of colonoscopy, provided a solution by creating colorectal diagnostic capacity and safe patient risk assessment.

CCE polyp detection was significantly higher than that for the gold standard, colonoscopy. Similar rates of patients with ≥ 5 polyps were recorded but there were twice as many ≥ 10-mm polyps detected at CCE 21 . Part of this may be explained by an overestimate of polyp size at CCE but this consistent finding suggests that CCE is detecting more polyps 22 23 24 25 . Double-counting seems an unlikely explanation because this was a “per-patient” finding. Data from systematic review and meta-analysis of tandem colonoscopy confirms that colonoscopy misses polyps > 10 mm in 9% and 6 to 9 mm in an additional 19% of cases 26 . The high-quality, standardized training of CCE readers may have contributed to high polyp detection at CCE. Published meta-analyses support the per-polyp and per-patient polyp detection sensitivity of CCE in the surveillance or screening population 3 4 27 28 . In future, improved polyp localization at CCE may support and improve colonoscopic lesion detection.

The large service evaluation conducted in Scotland, ScotCap, also included surveillance patients, but with a broader scope 29 . Here 53 of 193 surveillance patients (28%) required no further test following CCE, whereas 104 of 193 (54%) and 30 of 193 (16%) required a colonoscopy and flexible sigmoidoscopy, respectively. A deferred model for endoscopic intervention was not offered in ScotCap. The same group have recently described a missed cecal cancer 30 . In a surveillance cohort of 180 patients, Kroijer et al found that they could reduce colonoscopies by 43% when CCE was introduced as a filter test 31 . They had a similar overall polyp detection rate in complete and adequately prepared CCE of 69%. However, there were one-third fewer ≥ 10-mm polyps detected at onward colonoscopy. CCE had a similar sensitivity in a lower-risk screening population in those with a family history of colorectal polyps 32 .

There are significant methodologic and interpretive limitations to this study. It represents a pragmatic evaluation of consenting patients who were offered CCE within a pilot that had been introduced by the NHS as a response to the COVID-19 pandemic. Because each participating NHS site recruited patients as a means to evaluate the pilot, strictly enforced recruitment criteria were not applied. Because the primary aims of the study were to address the safety and accuracy of CCE, heterogeneity of patient selection was not immediately problematic, and for this reason, all recruited patients have been included. However, when considering the utility of CCE, caution is required. Any application of CCE as a colonoscopy-sparing triage tool in the surveillance setting is dependent upon polyp prevalence in that population. Not all patients recruited into this study strictly fulfilled the BSG/ACPGBI post-polypectomy guidelines that had been introduced into England in 2020 and subsequently endorsed by National Institute for Health and Care Excellence 7 33 . Over half of patients had an arguably lower surveillance risk and, therefore, were likely to have a lower polyp yield at surveillance. This is borne out by our observation that in the subgroup analysis of those fulfilling the guidelines, 48% went on to an immediate colorectal endoscopy and only 22% of patients were discharged. This compares with 44% and 29%, respectively, in the study as a whole. While each NHS site managed its own post-polypectomy surveillance program, it is anticipated that most would be expected to adhere to national guidelines; therefore, the utility of CCE in supporting colorectal diagnostic capacity creation needs to recognize this. In the recently published parallel NHS pilot that recruited symptomatic patients at intermediate risk of colorectal cancer, 84% of patients had their management modified by CCE 34 . In a capacity-constrained system, it may be that CCE is an unattractive option in the post-polypectomy surveillance setting. A health economic assessment is beyond the remit of this observational study and, post-COVID, the data are presented, largely, as a “proof of concept”. A conventional “back-to-back” CCE to colonoscopy design could not be applied in this study because of the imperative to provide additional diagnostic capacity within the COVID-19 recovery context; therefore, paired outcomes exist in fewer than half the patients recruited. Follow-up or registry data are not yet available for those whose onward interventions were deferred or who were discharged. Attempts to offset this, by the introduction of a comparator colonoscopy cohort, provides some degree of “false-negative” reassurance because prevalence of polyps was lower in the colonoscopy cohort. This proved to be a small cohort and is subject to selection bias; however, the older age and arguably higher surveillance risk in the colonoscopy cohort should have favored polyp detection in that group over the CCE cohort.

Completion and bowel preparation adequacy remain challenges for CCE. The headline figure of 62% is a concern but may give a slightly misleading impression because the “intention-to-investigate” polyp yield was higher than that seen in the comparator endoscopy cohort ( Table 1 ). What an incomplete and inadequately prepared CCE cannot do is exonerate the colon when normal and this resulted in 14% of all patients requiring immediate onward endoscopic investigation because of these quality difficulties. Many of these procedures were flexible sigmoidoscopies; however, 70% of them were in patients where the CCE quality prevented the EAG guidelines from being applied. The higher “normality rate” seen at flexible sigmoidoscopy suggests that many clinicians were taking a pragmatic approach to a poorly assessed left colon in an otherwise satisfactory CCE. There will also have been a “learning curve” here and a degree of clinical caution being exercised. CCE was a new modality to all in the pilot. Careful patient selection and information to support the patient are likely to improve the quality performance of CCE in future. Technological developments are likely to be developed soon to address lesion identification and description of lesions between CCE and colonoscopy 35 .

Colorectal diagnostics capacity may not be the challenge it was during COVID-19, although in England, we continue to fail to reach the Faster Diagnosis Standard for colorectal cancer 36 . Beyond this, there are many patient groups for whom colonoscopy is an unattractive option, such as those who are on anticoagulants or antiplatelet therapy or who had a painful prior experience of colonoscopy. Here CCE offers a safe and diagnostically accurate choice. Lower-risk surveillance cohorts and the tier system applied to CCE findings also profoundly influence the capacity created by the strategy described in this study. If Tier 1 management had been reserved solely for those with a polyp ≥ 10 mm, then only 17% of recruits would have gone on to immediate colorectal endoscopy. This is arguably a more clinically relevant scenario, recognizing now that we can be confident about the diagnostic accuracy of CCE.

This study purposely addressed patients due for a 3 yearly post-polypectomy colonoscopy because: 1) it represented the major group of surveillance patients; 2) the polyp risk could be defined from other datasets; and 3) a normal colonoscopy would close the surveillance episode for the patient. However, the findings from this study suggest that CCE could be extended into other surveillance settings where the discomfort of repeated colonoscopy can become onerous 37 .

## Conclusions

We conclude that in this large, clinically relevant study, CCE is safe and has a high diagnostic accuracy for colorectal polyps. Placed as a filter test with supportive management guidelines, CCE provides additional diagnostic capacity. However, that capacity release is more limited than in other patient groups, largely because of the high polyp yield in those undergoing surveillance. Nonetheless, CCE can sit well in the surveillance setting as a complementary service where patient choice or capacity constraints require an alternative to colonoscopy. This would allow endoscopic therapeutic resources to be made available to other colorectal pathways.
